# Public Health and Social Desirability in Kazakhstan: Methodological Considerations

**DOI:** 10.5195/cajgh.2015.191

**Published:** 2016-03-29

**Authors:** Brett J. Craig, Martha C. Engstrom

**Affiliations:** Department of Communication, Nazarbayev University, Kazakhstan

**Keywords:** public health, social desirability, control of health, Kazakhstan

## Abstract

**Background:**

As the Republic of Kazakhstan undertakes new public health efforts to promote healthy lifestyles among its citizens, the local perceptions of health and health behaviors need to be examined and understood from the sociocultural and historical perspectives. The primary aim of this study is to examine the association between perception of control on one’s health and engagement in good and bad health behaviors.

**Methods:**

Students enrolled in a health communication course surveyed 310 citizens of Kazakhstan on their perceptions of control over their own health and multiple health behaviors (i.e. smoking status, physical activity, etc.). Twenty-seven students were divided into groups and approached every third passerby as a potential participant during common shopping hours in nine popular marketplaces in Astana, Kazakhstan. Perception of control on one’s health was measured using a validated measure of health control: the multidimensional health locus of control scale (MHLC), developed by Wallston and colleagues. The MHLC measures three separate loci of control: internal, chance, and powerful others.

**Results:**

Participants perceived themselves as having highest control over their health (MHLC subscale internal: 29.70±0.64), powerful others had second highest control (MHLC subscale power others: 23.72±0.77), and chance had the lowest but still some control on their health (MHLC subscale chance: 20.82±0.85). Most participants rated their current health as very good (18.1%), good (45.0%), or moderate (32.3%). Approximately 23.4% of participants were smokers, and 22.2% consumed alcohol. Physical activity averaged 3.63 days in the past week, and fruit and vegetable consumption averaged 2 servings of each per day. Tobacco and the powerful others subscale were significantly negatively correlated (r=−0.17, *p*<0.05).

**Conclusions:**

Participant reports regarding personal health behaviors and lifestyle did not reflect the national reports regarding lifestyle behaviors. The relationship between powerful others subscale and tobacco smoking indicate that using healthcare providers may open up avenues to lowering tobacco use through patient education; however, social desirability is a notable concern for public health interventions. More importantly, the surveys uncovered future questions for conducting public health research with the general public, including issues of trust in the healthcare system and social desirability bias. Additional factors such as distrust in healthcare and government also may play a role in the public’s participation in social scientific research. The students who conducted the surveys reported a general skepticism from the public ranging from unfamiliarity with survey research to explicit distrust in the intentions and purpose of the research itself.

Health behaviors and public perception of health is an important area of investigation in the Republic of Kazakhstan. A previous study examining public perceptions of control over health and health behaviors faced methodological issues of survey research.^1^ Cultural and historical factors in Kazakhstan’s health services delivery likely influence the public’s reports on their individual health behaviors due to a focus on disease-centric healthcare rather than wellnesscentric.^2^ The purpose of this study was to examine the relationship between the general public’s perceptions of control of one’s health with health behaviors. This information can then be used to inform early efforts undertaken by the Kazakhstan government to promote healthy lifestyles.

## Health and the perception of control

Efforts in health promotion are generally structured around the general public’s assumptions regarding their current and future health status as related to or as a result of their health choices and behaviors.^3^ However, the strategies of health promotion using information dissemination or even social appeal are theoretical constructs that assume particular beliefs and perceptions about individual responsibility and control regarding outcomes.^2^ Research shows that different cultural groups demonstrate variance in health beliefs, health behaviors,^4^–^6^ and causes of illness.^7^ Furthermore, political backgrounds of healthcare systems contribute to the cultural perceptions of roles and responsibilities regarding healthcare professionals and the public.^8^

Health behaviors and beliefs have been studied extensively in the West, but in post-Soviet countries, this phenomenon is in need of further investigation.^1^,^9^ The post-Soviet Republic of Kazakhstan, in addition to undergoing significant changes in its healthcare model,^10^ is also beginning to develop efforts in public health promotion, which present healthy outcomes as a result of an individual’s health behaviors.^11^ Current public health intitiatives aim to address the increasing prevalence of lifestyle-related non-communicable diseases, specifically cardiovascular disease, in Kazakhstan. Health behaviors, such as smoking, are of particular interest. However, if the public does not see health outcomes as within their locus of control, current efforts in health promotion will not produce changes in behavior towards healthier lifestyles.

One established measure of perceived health locus of control is the multidimensional health locus of control scales (MHLC).^12^,^13^ The MHLC were developed by Wallston and colleagues to assess participants’ reports of how much control they perceive they have over their health. The MHLC contains three subscales: internal (whether you feel that you have control over your own health), chance (whether you feel your health is due to luck, fate, or chance), and powerful others (whether you feel that powerful individuals, such as physicians or other health professionals, control your health). Sample questions from these subscales are “If I take care of myself properly I can prevent diseases” (internal), “Most of the things that affect my health happened to me accidentally” (chance), and “The best way for me to avoid different sicknesses is to visit a doctor on a regular basis” (powerful others). There are 18 questions in the MHLC, six for each subscale, and participants are asked to respond on a six-item Likert scale from 1 (strongly disagree) to 6 (strongly agree). Scores range from 6 to 36.^5^,^12^,^13^ Each subscale score indicates the degree of control the participant attributes to that source. These three subscales are not mutually exclusive and can be reported as high in all, low in all, or at various levels. The higher the score, the more the participant is placing control in that source. Scores can vary across populations and groups, but in general participants who score above the median on any score can be considered “high” on that subscale, and those who score below could be called “low.”

## Cultural influences in survey research

The culture and social system of Kazakhstan is different than the context in which these measurement scales were derived, and the use of surveys among the general public is also a relatively new practice in Kazakhstan. Previous research has examined the influence of culture on survey research in areas such as social desirability, issues in translation and meaning, and the use of the Likert scale format.

Social desirability, using the definition of Johnson and Van de Vijver, is “the tendency of individuals to ‘manage’ social interactions by projecting favorable images of themselves, thereby maximizing conformity to others and minimizing the danger of receiving negative evaluations from them.”^14^ Though social desirability is likely to be a universal concept, a participant’s perception of which answer to a question would help him or her appear to be more socially desirable varies across cultures. Previous research has indicated that there are cultural influences on social desirability, particularly in collectivistic cultures, where survey respondents tend to answer questions in a manner that would be viewed favorably by others.^14^–^16^ Honesty with strangers in an interaction is valued more in individualist cultures, whereas a concern for social desirability can bias results to over-report good behavior or under-report bad behavior in collectivist cultures.^17^ Therefore, social desirability may be more of an influence in collectivist cultures than in individualist cultures.^18^,^19^

Effectively translating survey instruments from their source language is an important part of cross-cultural research. If meaning is not preserved across languages, the original intent of the instrument can be lost or distorted, in addition to compromising its validity and reliability.^20^ Embedded in language are the experiences and norms for word usage that can be different across cultures.^21^ In order to preserve meaning and intent, Brislin’s^22^ model of translation focuses primarily around the notion of back translation. An instrument should be translated to the operative language and then translated back to the source language by someone unfamiliar with the instrument in its source language. This way the back translation can be compared to the original instrument to check for consistent meanings.

The use of Likert scales, where participants respond by indicating on a scale the degree to which they agree or disagree with a statement, is very popular among instruments used in survey research. Research has shown people from different cultural groups tend to respond differently in surveys using Likert scales. Hui and Triandis^23^ found that some groups are more prone to report extremes on the scales than others, while Lee, Jones, Mineyama, and Zhang^24^ found that some groups favor the midpoint response when cultural beliefs would encourage it. Likert scales can include different quantities of items, but most common are 4-point, 5-point, 6-point, and 7-point. The MHLC scales used in this study are typically used with a 6-point scale response scale.

The objective of this paper is to establish the usability of the MHLC scale as well as explore the issues of survey research through street contacts with the general public in Kazakhstan. Researchers using these scales, as well as other similar public health measurements, may benefit from learning from the results of this study.

## Methods

To begin investigating how culture influences the perception of control and responsibility in health in Kazakhstan, surveys were distributed in nine marketplaces in Astana, Kazakhstan after receiving approval from Nazarbayev University’s research and ethics committee. Surveys included questions about basic demographic information and health behaviors, such as alcohol and tobacco use, exercise, and diet. We included the MHLC scales as the primary measure to determine levels of perceived control. Surveys were translated from their original language (English) into both Russian and Kazakh by a professional translator. A different professional translator who had not seen the original English version then back translated both translated versions into English. Modifications in the translated versions were then made to preserve the meaning and clarity of the original survey.

Students from the communication course *Science, Health and Social Influence*, a third-year course, participated in the collection of survey data for this study. After learning about the scales as a part of coursework, students were trained on how to approach participants and handle the distribution and collection of survey data. Their training took place as part of the course work and focused on the ethics of research, confidentiality, interaction with research participants, and techniques of answering questions about the research without biasing the responses. Twenty-seven students were divided into groups of three to create nine groups. Each group was assigned a popular marketplace within the city limits of Astana. The marketplaces and their corresponding locations within the districts are given in Table 1.

The marketplaces were chosen on the basis of improving regional diversity, while using marketplaces that represent common places of general public gathering. On a weekend during common shopping times, students approached every third passerby as a potential participant. This pattern of approaching participants did not create a truly random sample, but it helped prevent bias on the part of the students in whom they chose to approach. Though the marketplaces are commonly attended areas by many different groups of people, one limitation is that not all groups would be equally represented at the marketplaces, especially if their health conditions do not permit such activity. Records of completed surveys and the numbers of potential participants approached were carefully monitored. Students were asked to record their experiences and impressions afterwards to provide further data on this pilot study.

### Data analysis

First, we conducted descriptive statistics on participant characteristics. Second, we examined the association of participant characteristics (e.g. age, sex, etc.) on MHLC sub-scales using independent-samples t-tests. Finally, we used bivariate correlation to test the association between participant characteristics and each of the MHLC sub-scales. All analyses were conducted using SPSS, using *p*<0.05 as the cut-off for significance.

## Results

### Participants

Students collectively approached 824 people, 310 of which agreed to participate, yielding a 38% survey response rate. Of those who responded, 57.7% were female with a mean age of 32.53 (*SD*=13.65). Details regarding the participant language, ethnicity, and marital status can be found in Table 2.

### Descriptives of participant health

Participants were asked to rate their overall health status. Nearly all participants rated their current health as moderate (32.3%), good (45.8%), or very good (18.1%). With regards to health behaviors, 23.4% of participants were smokers and 22.3% consumed alcohol. Physical activity averaged around 3.63 days in the past week, and fruit and vegetable consumption averaged 2 servings of each per day. Further information on participant health ratings and reported behaviors can be found in Table 3.

### Multidimensional health locus of control scores

Most participants tended to perceive themselves as having high control over their health (MHLC subscale internal: 29.70±0.64). Participants perceived others, such as medical providers, as having high control over their health (MHLC subscale power others: 23.72±0.77). Participants perceived chance as a somewhat likely entity to have control over their health (MHLC subscale chance: 20.82±0.85).

### Analysis of association and correlation of predictors of MHLC subscales

Most health behaviors were not significantly correlated with any of the reported MHLC subscales and differences in sex, age, language or ethnicity. Tobacco smoking was significantly, though weakly, associated with the powerful others subscale (r=−0.17, *p*<0.05).

## Discussion

In this study, in addition to the results of the MHLC survey, we found methodological issues common to cross-cultural research, such as translation of measurements and social desirability in addition to other issues perhaps more unique to the region.

### MHLC results

Such MHLC scores are different than white European cultural groups, but they are similar to South Asian cultural groups, especially with the powerful others and internal subscales,^5^ revealing possible cultural influences on perceptions of health and health behaviors. The subscales, developed in a Western cultural setting, often find that those reporting a high level of internal control report lower scores in control residing in external areas such as chance or powerful others. However, as Steptoe and Wardle^3^ found in their study of Eastern Europeans and Wrightson and Wardle^6^ found in their study comparing white Europeans with South Asian and Afro-Caribbean, these subscales are not always at odds with one another in other cultural groups.

Our pilot study revealed that this also might be the case in Kazakhstan. While these participants reported high levels of internal control, they also reported high levels of control coming from powerful others (influential people in their lives). Some possible explanations of this perception include the influence of the Soviet model of healthcare that focused on treatment of disease rather than on health and prevention.^1^,^25^ Healthcare was (and largely still is) provided by the government and, therefore, was seen as the responsibility of the government. While healthy lifestyles are now being promoted and the public may even be reporting a sense of control over their own health through their lifestyle, the perception that health still resides in the hands of powerful others such as healthcare providers may be influencing the powerful others subscale score.

The significant negative correlation between tobacco use and the powerful others subscale also deserves further investigation. Those who are using tobacco are less likely to perceive healthcare providers as influencing their health, while those who perceive healthcare providers as having some control over their heath are less likely to use tobacco. Possible explanations for this phenomenon include anti-tobacco messages from healthcare providers as well as those participants who are generally more health conscious and avoid tobacco also see their healthcare providers regularly.

### Methodological issues

Though several methodological features of this study were consistent with similar studies done elsewhere such as the response rate of street contacts and the influence of survey fatigue, by using the reported experiences of students distributing the surveys we found that the issues of translation, Likert scales, and social desirability may be significantly influencing the effectiveness of using surveys, particularly through street contacts. Furthermore, the additional issue of trust was made salient in the interactions between participants and survey distributors.

### Translation and meaning

Though we had the survey translated and back translated into both Russian and Kazakh languages so that participants could respond in their native language, participants struggled with many of the questions and some were irritated with the translation. Students distributing these surveys reported most frequently that participants made comments about the questions in the MHLC scales being repetitive and not discernibly different from one another. This may be an imperfection in accurately translating subtle differences in the questions, and it may also reveal differences in constructions of concepts and perceptions embedded within the languages themselves regarding relationships with others and control over situations and outcomes.

As the government of Kazakhstan has been strengthening the use and presence of the Kazakh language,^26^ the need for properly translated instruments will continue to grow. As demonstrated, translation is extremely important for linguistic comprehension and for cultural conceptualization and familiarity.

### Likert scales and time

The use of Likert scales was not reported by participants as a point of confusion; however, the unfamiliarity with the concept as well as using the 6-point scales may have contributed to time and even survey fatigue. Many students noted that it took much longer than typical to fill out a two-page questionnaire. Other research suggests simplifying a 6-point to a 4-point scale for different populations without a loss in reliability or validity.^20^ The confusion created by the fact so many questions seemed to be the same according to the participants might have been compounded by the choice in six responses for each question. Using 4-point scales or more simplified versions of responses may improve the accuracy of responses and decrease survey fatigue.

### Social desirability and culture

The participants’ reports on tobacco and alcohol use are much lower than expected considering the World Health Organization’s (WHO) nationwide statistics on usage.^27^ Additionally, their reports on diet and exercise indicate a much healthier sample than the greater population where the prevalence of behaviorally related diseases creates a significant burden and is projected to increase.^28^ Though our sample of participants is not truly random and cannot be expected to closely match generalized figures, the relatively healthy behaviors reported on average indicate the possibility of social desirability influencing responses. As Kazakhstan is a more collectivistic than individualistic society, it is likely that many survey responses may be influenced by a strong desire to save face, especially when answers to questions can reflect a negative image. Some students reported experiences where participants either ignored or did not understand anonymity and privacy and felt they had to explain and justify each of their answers to the students. A few students rather indignantly reported that they knew participants were not being honest about their behaviors such as tobacco use because students could see packages of cigarettes in participants’ hands or pockets. However, the age of the students needs to be considered in the collection of data because Kazakhstan is a hierarchical culture which values age as well as position. Thus, their responses to these students may be influenced by these norms and values.

If social desirability significantly influenced these participants’ responses, this may further add to the knowledge gained from this pilot study. In order for social desirability to influence participants’ answers regarding health behaviors, the participants must possess a basic knowledge of what a healthy lifestyle is. Therefore, though their health behaviors may not actually be as healthy as they reported, participants may have more knowledge of what healthy behaviors are and why they are socially desirable than was expected. If health promotion is to be effective it needs to be informed by research that has examined influences on health behaviors beyond knowledge and information. Research in Kazakhstan needs to explore social and economic factors that are shaping the population’s health behaviors. More research is needed regarding the public’s perspectives on why they do what they do rather than simply what they know.

### Trust

One unanticipated issue students encountered in administering questionnaires was lack of trust in the healthcare system. Participants frequently asked about the purpose of the survey. When students told them it was to help better promote health in Kazakhstan, they generally expressed disbelief. Some participants laughed and mocked the students for being naïve. Others refused to participate because it would be a “waste of time.” Some participants shared stories of negative experiences with healthcare system, while others went so far as to name doctors who they felt were responsible for bad care. Additionally, participants expressed distrust in the research process itself, claiming such work will not help improve anything. However, some participants were interested in knowing the results of the survey and even expressed appreciation, saying more research like this needed to be done.

Trust is a common issue in many post-Soviet healthcare systems because of Soviet-era characteristics, such as paternalism, that current governments are struggling to change. Health care during the Soviet era was largely underfunded and included non-evidence based practices and beliefs,^8^ where corruption has been a problem in the form of informal payments.^29^ These characteristics, coupled with lack of knowledge on preventive practices, results in the general public not seeking timely medical care.^30^ The perception that utilizing healthcare services will not yield desired results needs to be addressed in future research. The issue of trust may have higher relevance when considering the high MHLC subscale score of powerful others. If the public perceives control over their health to be in the hands of powerful others such as doctors but does not trust doctors, this may influence health behaviors and choices leading to negative outcomes. It may also dissuade the public from using medical services in appropriate and necessary ways.

One of the most important methodological approaches utilized in this study was the involvement of students in data collection. We believe that this experience exposed students to the nature of research and data collection and helped them to think more critically about data collection and statistical approaches to use for survey research. It also helped students to see the value in seeking out and measuring the public’s perception because through their experiences these students learned how diverse in opinion and experience their fellow citizens actually are. While we attempted to obtain a diverse sample of participants in this study by going to marketplaces belonging to different socioeconomic classes, we also acknowledge the limitations in the generalizability of this study. Astana itself as the capital city of Kazakhstan is drawing people from different regions within the country to live there, and as such may not be representative of the entire country.

## Conclusions

This pilot study has revealed significant challenges to conducting face-to-face survey research with the general population of Kazakhstan. Due to cultural influences on social interactions with research staff, participants’ answers to survey questions could be significantly influenced by social desirability. Additionally, a history of distrust with authority figures may also influence participants to alter their answers or influence decision to participate in research. These are issues that need to be considered and accounted for in designing and carrying out social scientific research in Kazakhstan, and Central Asia in general.

## Figures and Tables

**Table 1:** Marketplaces in Astana by district

Market	District
Tsum	Saryarka West
Alem	Saryarka North
Khan Shatyr	Yesil West
Near railway station	Saryarka outer region
New Central Market	Almaty North
Anvar Food Fair	Yesil South
Arteom	Saryarka Center
Gulzhan	Saryarka Center
Shapagat	Saryarka East

**Table 2:** Demographic descriptive characteristics of participants

Variables	m±SD or N(%)
Age	32.53 ± 13.65
Sex	
Male	130 (41.9)
Female	179 (57.8)
Unknown	1 (0.3)
Language	
Russian	231 (74.5)
Kazakh	79 (25.5)
Ethnicity	
Kazakh	230 (74.2)
Russian	51 (16.5)
Other/Unknown	29 (9.3)
Marital status	
Currently married	131 (42.3)
Never married	108 (34.8)
Divorced	19 (6.1)
Widowed	13 (4.2)
Cohabitating	10 (3.2)
Separated	5 (1.6)
Cohabitating	2 (0.6)
Unknown	22 (7.2)

**Table 3.** Descriptive characteristics of participant health

Variables	Total m±SD or N (%)	Female m±SD or N (%)	Male m±SD or N (%)
Physical activity	3.63 ± 0.23	3.08 ± 2.85	3.83 ± 2.63
Fruit servings	2.03 ± 0.23	2.08 ± 1.86	1.96 ± 2.02
Health status rating			
Very good	56 (18.1)	22 (12.2)	34 (26.2)
Good	142 (45.8)	84 (46.9)	58 (44.6)
Moderate	100 (32.3)	66 (36.9)	34 (26.2)
Bad	3 (1.0)	3 (1.7)	0 (0.0)
Very bad	1 (0.3)	1 (0.6)	0 (0.0)
Unknown	8 (2.5)	3 (1.7)	4 (3.0)
Tobacco smoking			
Daily	37 (12.0)	6 (3.4)	31 (23.8)
Non-daily	32 (10.3)	13 (7.3)	19 (14.6)
Did not smoke	224 (72.3)	151 (84.3)	73 (56.2)
Unknown	17 (5.4)	9 (5.0)	7 (5.4)
Alcohol consumption (past week)			
None	215 (69.3)	118 (65.9)	97 (74.6)
Yes (average of 2.28 drinks)	69 (22.3)	36 (20.1)	33 (25.4)
Unknown	26 (8.4)	25 (14.0)	0 (0.0)
